# Age at period cessation and trajectories of cardiovascular risk factors across mid and later life

**DOI:** 10.1136/heartjnl-2019-315754

**Published:** 2020-02-25

**Authors:** Linda Marie O'Keeffe, Diana Kuh, Abigail Fraser, Laura D Howe, Debbie Lawlor, Rebecca Hardy

**Affiliations:** 1 MRC Integrative Epidemiology Unit, University of Bristol, Bristol, UK; 2 Population Health Science, Bristol Medical School, Bristol, UK; 3 School of Public Health, University College Cork, Cork, Ireland; 4 MRC Unit for Lifelong Health and Ageing at UCL, London, UK; 5 CLOSER, UCL Institute of Education, London, UK

**Keywords:** epidemiology, cardiac risk factors and prevention

## Abstract

**Objective:**

To examine the association between age at period cessation and trajectories of anthropometry, blood pressure, lipids and glycated haemoglobin (HbA1c) from midlife to age 69 years.

**Methods:**

We used data from the UK Medical Research Council National Survey of Health and Development to examine the association between age at period cessation and trajectories of systolic blood pressure (SBP), diastolic blood pressure (DBP), body mass index (BMI) and waist circumference (WC) from 36 to 69 years and trajectories of triglyceride, low density lipoprotein cholesterol (LDL-C), high density lipoprotein cholesterol (HDL-C) and HbA1c from 53 to 69 years.

**Results:**

We found no evidence that age at period cessation was associated with trajectories of log triglyceride, LDL-C and HDL-C from 53 to 69 years and trajectories of SBP or DBP from 36 to 69 years, regardless of whether period cessation occurred naturally or due to hysterectomy. While we found some evidence of associations of age at period cessation with log BMI, log WC and log HbA1c, patterns were not consistent and differences were small at age 69 years, with confidence intervals that spanned the null value.

**Conclusion:**

How and when women experience period cessation is unlikely to adversely affect conventional cardiovascular risk factors across mid and later life. Women and clinicians concerned about the impact of type and timing of period cessation on conventional cardiovascular intermediates from midlife should be reassured that the impact over the long term is small.

## Introduction

A recent systematic review and meta-analysis of observational studies showed that age at period cessation (natural and surgical combined) before 45 years was associated with greater coronary heart disease (CHD) mortality, cardiovascular disease (CVD) mortality but not stroke mortality.^1^ Since that review, several other large studies have supported these findings,^2–4^ with one study suggesting a weaker association for age at surgical menopause than for age at natural menopause.^2^ Evidence for an inverse association of age at natural menopause and CVD risk is also supported by recent findings from a Mendelian randomisation study that showed that genetic variants associated with earlier age at natural menopause were associated with increased CVD risk in women.^5^

In contrast to the large body of literature demonstrating inverse associations between age at period cessation and CVD risk and mortality, the aforementioned systematic review^1^ highlighted a lack of studies focusing on age at period cessation and intermediate risk factors such as body mass index (BMI), blood pressure and diabetes. Available longitudinal studies with repeated measures of CVD risk factors across midlife, such as the Study of Women’s Health across the Nation (SWAN) and Coronary Artery Risk Development in Young Adults (CARDIA)^6–8^ focused on acute and immediate changes close to the menopausal transition rather than examining whether age at period cessation was associated with change over the long term.^6–8^ The few available studies that have examined intermediate risk factors have largely demonstrated a lack of association between age at period cessation and BMI, waist hip ratio, diastolic blood pressure (DBP), systolic blood pressure (SBP), glucose, risk of diabetes, hypertension and obesity 9–13; however, these studies have mostly been cross-sectional and compared different groups of pre-and post-menopausal women, often with only a single measure of the intermediate risk factor and retrospective reports of age at period cessation in women who were already post-menopausal. Thus, prospective analyses of age at period cessation and change in CVD risk factors across midlife are required to clarify the association between age at period cessation and conventional CVD intermediates from midlife to older age; such analyses go beyond examination of age at period cessation and outcomes at a single time point, providing insights into whether trajectories of CVD risk factors from mid to later life differ by age at period cessation.

We examined the association between age at period cessation, by type of period cessation (hysterectomy compared with natural menopause) and five repeated measures of blood pressure, BMI and waist circumference (WC) from age 36 to 69 years, and three measures of lipids and glycated haemoglobin (HbA1c) from age 53 until age 69 years, in the UK Medical Research Council (MRC) National Survey of Health and Development (NSHD).

## Methods

### Participants

The MRC NSHD is a socially stratified sample of 5362 subjects (2547 females and 2815 males) followed 24 times since birth in England, Scotland and Wales in the first week of March 1946, with an additional nine separate postal questionnaires sent to women in midlife.^14 15^ Ethical approval for the most recent visit when participants were aged 69 years was given by Queen Square Research Ethics Committee (13/LO/1073) and Scotland A Research Ethics Committee (14/SS/1009). Participants provided written informed consent for each visit.

### Data

#### Blood pressure and anthropometry

SBP, DBP, height, weight and WC were measured at 36, 43, 53, 60–64 and 69 years. Blood pressure was measured at least twice at 36 and 43 years using a Hawksley Random Zero sphygmomanometer^16^ and three times at ages 53, 60–64 and 69 years using an Omron HEM-705, while the study member was seated and after a short period of rest.^17^ Measurements from the Random Zero sphygmomanometer were adjusted using published conversion equations to achieve compatibility with later measurements.^18^ The second blood pressure reading was used unless it was missing, in which case the first was used. Height (cm), weight (kg) and waist (cm) were measured according to a standardised protocol at all home visits and the research clinic. BMI was calculated by dividing weight in kg by height in metres squared (kg/m^2^).

#### Lipids and HbA1c

Blood based markers (triglycerides, low density lipoprotein cholesterol (LDL-C), high density lipoprotein cholesterol (HDL-C) and HbA1c were measured at 53, 60–64 and 69 years. A venous blood sample was taken by the nurse according to a standardised protocol at ages 53 (non-fasting),^19^ 60–64 (fasting)^14^ and 69 years (non-fasting).^15^ Total cholesterol was measured by enzymatic CHOD–PAP (cholesterol oxidase/peroxidase aminophenazone). Triglycerides were measured using a glycerol/kinase POD linked reaction of glycerol liberated enzymatically from triglycerides; LDL-C was calculated using the Friedewald formula, and precipitation for measurement of HDL-C was performed using phosphotungstic Mg^2+^. All these measurements were made with a Bayer DAX-72. Samples were analysed for glycated haemoglobin with the Tosoh A1C 2.2 Plus Analyser (Tosoh, Tokyo, Japan) using high performance liquid chromatography.

#### Type and timing of menopause

Information on menstrual irregularity, month and year of last menstrual cycle or any operation to remove the uterus or ovaries and monthly hormone replacement therapy (HRT) use was obtained from annual postal questionnaires between the ages of 47 and 54 years (inclusive), with an additional one at 57 years, and from the face to face interviews with nurses at 43, 53 and 60–64 years.^20 21^ Age at which periods ceased naturally (defined as a period of at least 12 months without menstruation) or because of bilateral oophorectomy (with or without hysterectomy), or because of hysterectomy with or without unilateral oophorectomy was calculated. We excluded women whose periods stopped for other reasons, such as chemotherapy (n=37). We also excluded 189 women starting HRT before menopause who had not ceased HRT for at least a year before giving responses about period regularity and the timing of the last period because it was not possible to assign an accurate date of menopause among these women.

#### Potential confounders

We considered the following as potential confounders of the association between age at period cessation and CVD risk factors: socioeconomic position, parity, HRT use throughout follow-up, age at menarche, smoking at 36 years and physical activity at age 36 years. BMI at 36 years was considered as an additional confounder for blood pressure, lipids and HbA1c. The 36 year measures were selected for BMI, smoking and physical activity because these measures represent pre-menopausal measures for most women. Further information can be found in supplementary eAppendix 1.

10.1136/heartjnl-2019-315754.supp1 Supplementary data

### Sample sizes

Of the original birth cohort of females (n=2547), 1666 women were eligible for inclusion in analyses of blood pressure and anthropometry and 1563 women were eligible for inclusion in analyses of lipids and HbA1c. Of these women, only participants with a known date of period cessation, at least one measure of the risk factor and complete data on all confounders were included in analyses. Supplementary eFigure 1 and eTable 1 provide an overview of the study design and measures available.

### Modelling of age-related change in risk factors

We used multilevel models to examine change over time in CVD risk factors,^22^ as performed previously in other cohorts.^23–25^ Multilevel models estimate mean trajectories of the outcome (here CVD risk factors) while accounting for the non-independence (ie, clustering) of repeated measurements within individuals, change in scale and variance of measures over time and differences in the number and timing of measurements between individuals, using all available data from all eligible participants under a Missing at Random (MAR) assumption that the value of the missing risk factor can be predicted by other measured variables.^26^ Changes in triglyceride, LDL-C, HDL-C and HbA1c were modelled using a linear age term (two levels: measurement occasion and individual), allowing risk factors to change linearly from 53 to 69 years. Linear spline multilevel models (two levels: measurement occasion and individual) were used to model change in SBP, DBP, BMI and WC. Linear splines allow knot points to be fitted in order to derive different periods in which change is approximately linear. In this analysis, we fit a knot point at age 53 years resulting in two periods of change for SBP, DBP, BMI and WC from 36 to 53 years and from 53 to 69 years. All trajectories were modelled in MLwiN version 2.36,^27^ called from Stata version 14^28^ using the runmlwin command.^27^

Values of CVD risk factors that had a skewed distribution (BMI, WC, triglyceride, HBA1c) were (natural) log transformed before analysis. Further details of model selection are provided in the supplementary material (supplementary eAppendix 2) and model fit statistics are provided in supplementary eTables 2 and 3.

We examined the association of age at period cessation and type of period cessation (hysterectomy compared with natural menopause) with trajectories of each risk factor. We included variables for type of period cessation and age at period cessation and their interaction with age. We also included an interaction term for age at period cessation and type of period cessation to examine whether the association of age at period cessation differed by type of period cessation. Age at period cessation was centred at the mean age of period cessation for the sample (age 50 years). We also performed several additional and sensitivity analyses. Further details of these analyses can be found in the supplementary material (supplementary eAppendix 3).

## Results

Of the 908/915 women included in the analyses of blood pressure and anthropometry, most women were pre-menopausal at age 36 years, when the first measure of these was available (99.6% of women who had a natural menopause and 89.6% of women who had a hysterectomy were pre-menopausal at this age). Of the 787 women included in analyses of lipids and HbA1c, 94% of women who underwent a hysterectomy had the surgery by 53 years and 61% of women who underwent a natural menopause were post-menopausal by age 53 years. Women who had a hysterectomy were more likely to be in a lower social class, have higher parity, higher prevalence of current smoking at age 36, higher prevalence of physical inactivity at age 36, higher prevalence of HRT use across all time points and lower mean age at menarche and age at period cessation compared with women who had a natural menopause (table 1).

**Table 1:** Characteristics of participants included in primary analyses of anthropometry by menopause type (n=915)

	Natural menopause n=675 (n=672, 99.6% pre-menopausal at age 36 years (first measure of WC/BMI))	Hysterectomy n=240 (n=215, 89.6% pre-menopausal at age 36 years (first measure of WC/BMI))
	n (%)	n (%)
Household social class		
Professional/intermediate	256 (37.9)	76 (31.7)
Skilled (non-manual)	241 (35.7)	80 (33.3)
Skilled manual and partly skilled	132 (19.6)	70 (29.2)
Unskilled	46 (6.8)	14 (5.8)
Parity		
0	100 (14.8)	15 (6.3)
1 or 2	379 (56.2)	130 (54.2)
3 or more	196 (29.0)	95 (39.6)
Current smoking at age 36	233 (34.5)	91 (37.9)
Physical activity at age 36		
Inactive	275 (40.7)	110 (45.8)
Less active	167 (24.7)	53 (22.1)
Most active	233 (34.5)	77 (32.1)
HRT use		
Age 36	4/652 (0.6)	5/235 (2.1)
Age 43	15/623 (2.4)	17/228 (7.5)
Age 53	91/512 (17.8)	101/174 (58.1)
Age 60–64	9/498 (1.8)	22/173 (12.7)
Age 69	8/465 (1.7)	8/168 (4.8)
Mean age at menarche (SD)	13.1 (1.3)	12.9 (1.1)
Mean age at period cessation (SD)	51.5 (3.9)	44.1 (6.2)

BMI, body mass index; HRT, hormone replacement therapy; WC, waist circumference.

Women included in analyses were likely to be in a higher social class, have lower smoking prevalence at age 36, lower HRT use from 53 to 69, and have undergone a hysterectomy compared with women excluded from analyses due to missing exposure, confounder and outcome data. However, women included in analyses did not differ in parity, physical activity, mean age at menarche, mean age at period cessation, mean BMI at age 36 or mean SBP at age 36 compared with women excluded from analyses (supplementary eTable 4).

### Age at period cessation

**Figure 1:**
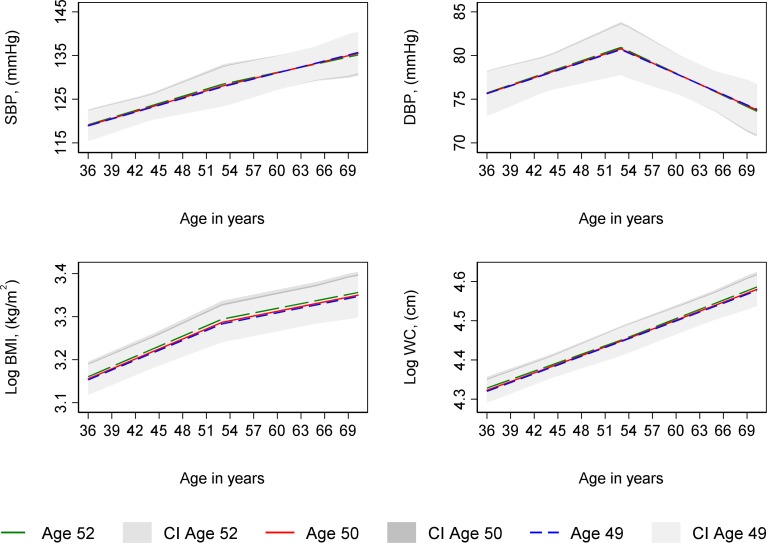
Mean predicted confounder adjusted trajectories of SBP, DBP, log BMI and log WC from 36 to 69 years, by age at natural menopause. BMI and WC are natural log transformed. Trajectories adjusted for socioeconomic position, parity, time-varying hormone replacement therapy use, age at menarche, BMI at age 36 (SBP and DBP only), smoking at age 36, and physical activity at age 36. Trajectories for the 75th (age 52, green line), median (age 50, red line) and 25th percentile (age 49, blue line) of age at period cessation among women with a natural menopause. BMI, body mass index; CI, 95% confidence interval; DBP, diastolic blood pressure; SBP, systolic blood pressure; WC, waist circumference.

**Figure 2:**
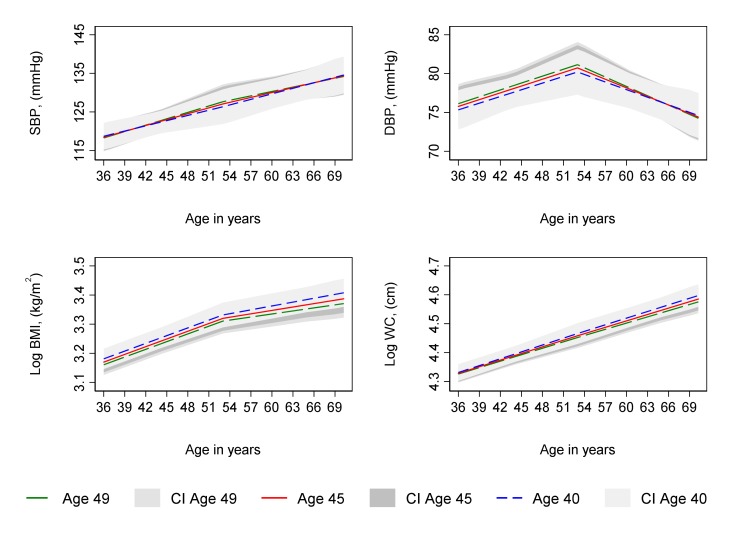
Mean predicted confounder adjusted trajectories of SBP, DBP, log BMI and log WC from 36 to 69 years, by age at hysterectomy. Trajectories adjusted for socioeconomic position, parity, time-varying hormone replacement therapy use, age at menarche, BMI at age 36 (SBP and DBP only), smoking at age 36, and physical activity at age 36. Trajectories for the 75th (age 49, green line), median (age 45, red line) and 25th percentile (age 40, blue line) of age at period cessation among women with hysterectomy. BMI, body mass index; CI, 95% confidence interval; DBP, diastolic blood pressure; SBP, systolic blood pressure; WC, waist circumference.

Age at period cessation was not associated with SBP and DBP at age 36 and change in these from 36 to 69 years in unadjusted (supplementary eTable 5) and confounder adjusted analyses (supplementary eTable 6 and figures 1 and 2). In unadjusted analyses (supplementary eTable 5), a 1 year older age at natural menopause was associated with higher log WC at age 36 years although with confidence intervals that spanned the null value; there was no evidence that older age at natural menopause was associated with change in log WC from 36 to 69 years and the difference at age 36 persisted at age 69 years, although with confidence intervals that spanned the null value. Conversely, a 1 year older age at hysterectomy was associated with lower log WC at 36 and lower log WC at age 69 years, though with confidence intervals that spanned the null value. The same patterns of association were observed between age at period cessation and log BMI at ages 53 and 69 years, although associations were weaker. Findings for log WC and log BMI were similar in confounder adjusted analyses (supplementary eTable 6 and figures 1 and 2).

**Figure 3:**
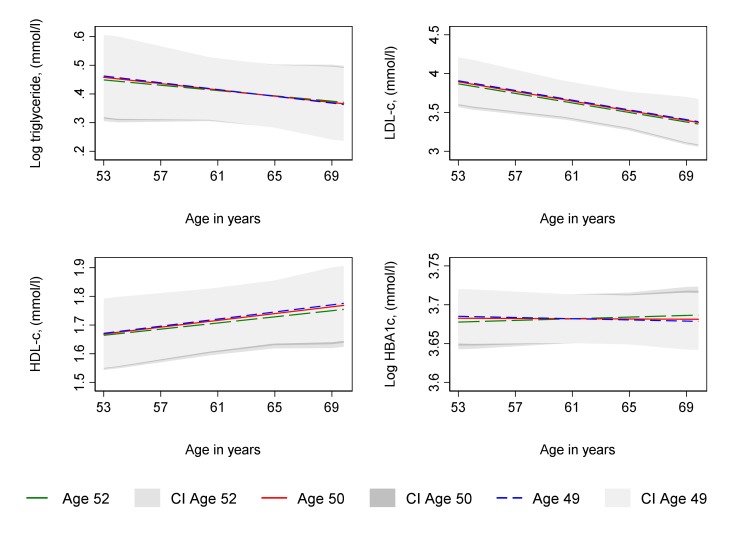
Mean predicted confounder adjusted trajectories of lipids and HbA1c from 53 to 69 years, by age at natural menopause. HbA1c and triglyceride are natural log transformed. Trajectories adjusted for socioeconomic position, parity, time-varying hormone replacement therapy use, age at menarche, BMI at age 36, smoking at age 36, and physical activity at age 36. Trajectories for the 75th (age 52, green line), median (age 50, red line) and 25th percentile (age 49, blue line) of age at period cessation among women with a natural menopause. BMI, body mass index; CI, 95% confidence interval; HbA1c, glycated haemoglobin; HDL-C, high density lipoprotein cholesterol; LDL-C, low density lipoprotein cholesterol.

**Figure 4:**
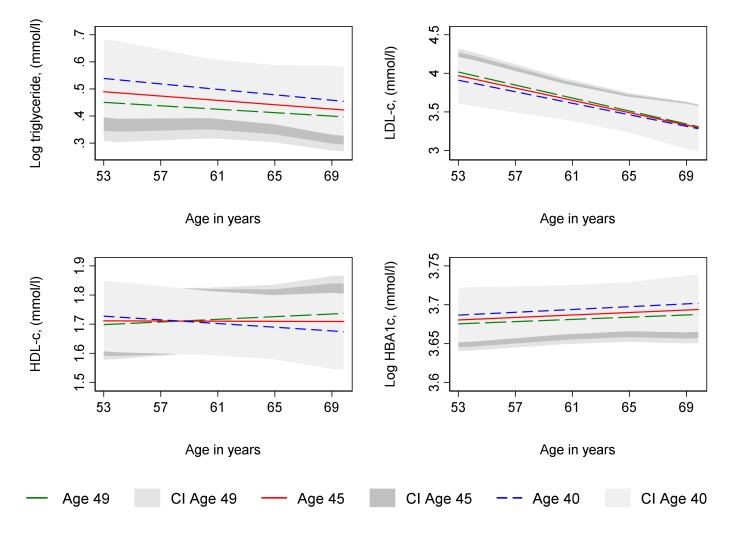
Mean predicted confounder adjusted trajectories of lipids and HbA1c from 53 to 69 years, by age at hysterectomy. HbA1c and triglyceride are natural log transformed. Trajectories adjusted for socioeconomic position, parity, time-varying hormone replacement therapy use, age at menarche, BMI at age 36, smoking at age 36, and physical activity at age 36. Trajectories for the 75th (age 49, green line), median (age 45, red line) and 25th percentile (age 40, blue line) of age at period cessation among women with hysterectomy. BMI, body mass index; CI, 95% confidence interval; HbA1c, glycated haemoglobin; HDL-C, high density lipoprotein cholesterol; LDL-C, low density lipoprotein cholesterol.

Age at period cessation, whether due to natural menopause or hysterectomy, was not associated with log triglyceride, LDL-C and HDL-C from age 53 and change in these from 53 to 69 years in unadjusted (supplementary eTable 7) or confounder adjusted analyses (supplementary eTable 8 and figures 3 and 4). In unadjusted analyses (supplementary eTable 7), age at natural menopause and age at hysterectomy were each associated with lower log HbA1c at age 53 years, although with confidence intervals that spanned the null value. However, a 1 year older age at natural menopause was associated with a faster increase in log HbA1c from 53 to 69 years such that by 69 years, older menopause was associated with higher log HbA1c, although confidence intervals spanned the null value. In contrast, older age at hysterectomy was associated with slightly slower increases in log HBA1c from 53 to 69 leading to lower log HBA1c at age 69 years, although with a confidence interval that spanned the null value. Findings for log HbA1c were similar in confounder adjusted analyses (supplementary eTable 8 and figures 3 and 4).

### Type of period cessation

**Figure 5:**
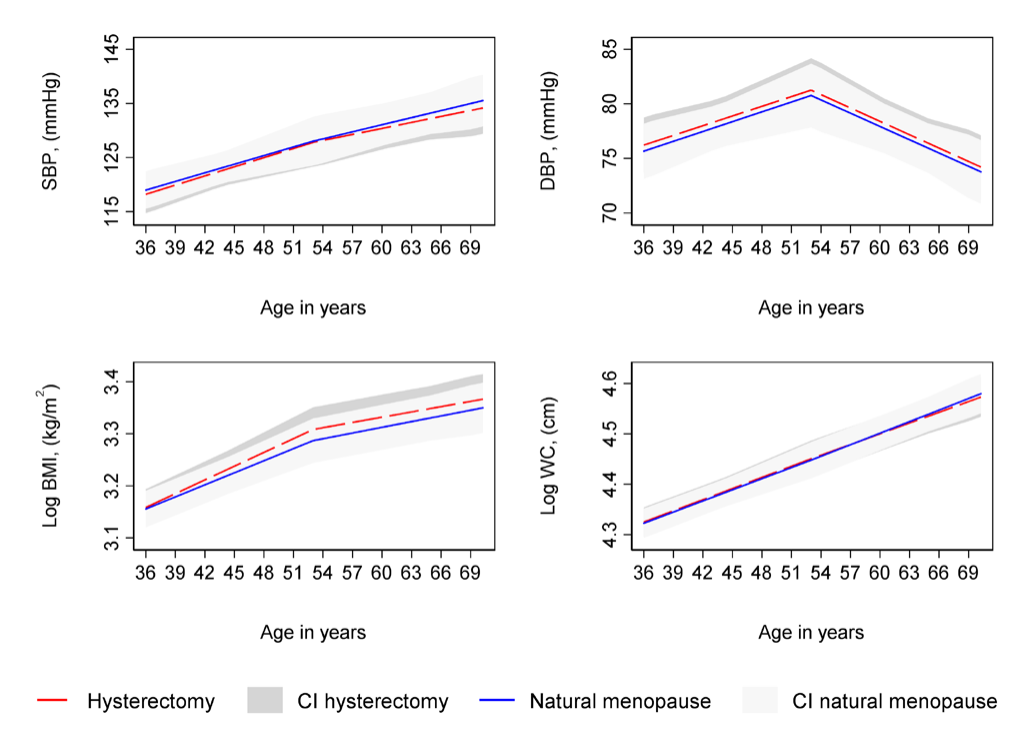
Mean predicted confounder adjusted trajectories of SBP, DBP, log BMI and log WC from 36 to 69 years, by type of period cessation. Trajectories adjusted for socioeconomic position, age at period cessation, parity, time-varying hormone replacement therapy use, age at menarche, BMI at age 36 (SBP and DBP only), smoking at age 36, and physical activity at age 36. BMI, body mass index; CI, 95% confidence interval; DBP, diastolic blood pressure; SBP, systolic blood pressure; WC, waist circumference.

**Figure 6:**
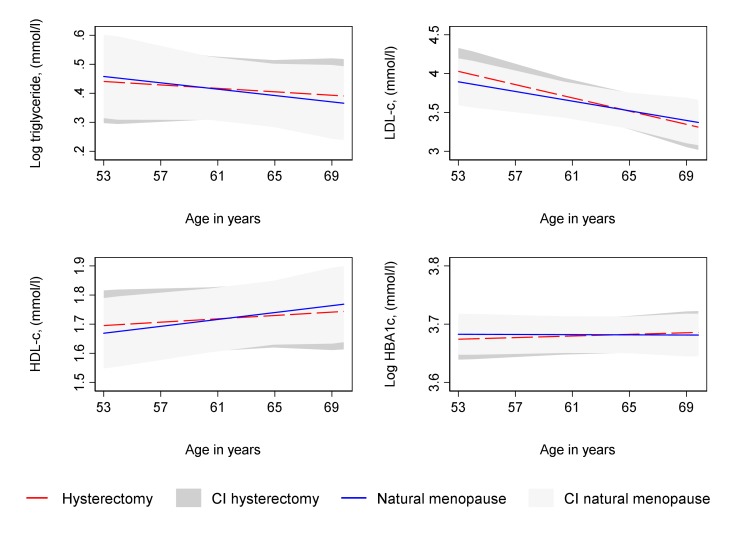
Mean predicted confounder adjusted trajectories of lipids and HbA1c from 53 to 69 years, by type of period cessation. HbA1c and triglyceride are natural log transformed. Trajectories adjusted for socioeconomic position, age at period cessation, parity, time-varying hormone replacement therapy use, age at menarche, BMI at age 36, smoking at age 36, and physical activity at age 36. BMI, body mass index; CI, 95% confidence interval; HbA1c, glycated haemoglobin; HDL-C, high density lipoprotein cholesterol; LDL-C, low density lipoprotein cholesterol.

Type of period cessation (hysterectomy compared with natural menopause) was not strongly associated with trajectories of SBP, DBP, log BMI and log WC from 36 to 69 years (supplementary eTable 9 and figure 5) in unadjusted and confounder adjusted analyses or with log triglyceride and LDL-C from 53 to 69 years (supplementary eTable 10 and figure 6). In unadjusted analyses, there was some evidence of faster increases in log HbA1c and faster decreases in HDL-C from 53 to 69 years among women who had a hysterectomy (supplementary eTable 10); however, these differences attenuated upon adjustment for confounders (supplementary eTable 10 and figure 6).

### Additional and sensitivity analyses

We found little evidence that the association of age at hysterectomy with any of the CVD risk factors varied by type of procedure (conservation of at least one ovary or bilateral oophorectomy). Our findings were not altered when we accounted for treatment of lipids, HbA1c and blood pressure. In sensitivity analyses excluding women who had reached menopause after 53 years, findings for the association of age at period cessation or type of period cessation with CVD risk factors were not appreciably altered compared with our primary analyses; all these results are available on request from the authors.

## Discussion

In a British birth cohort with prospective assessment of age at period cessation and repeated measures of eight conventional CVD intermediates from 36/53 to 69 years, we found little evidence of associations between age at period cessation or type of period cessation and trajectories of anthropometry and blood pressure (from 36 years) and lipids and HbA1c (from 53 years) up to age 69 years.

### Comparison with other studies

A lack of studies focusing on intermediate CVD risk factors was highlighted in the most recent systematic review of age at period cessation and CVD.^1^ Our findings are comparable with the few available studies of intermediate CVD risk factors included in that review. For example, in a cross-sectional study of Chinese women aged 40 to 59 years (n=2498 post-menopausal women and n=2245 pre-menopausal women), age at natural menopause was not associated with any intermediate risk factors studied, including risk of hypertension, diabetes, dyslipidaemia and obesity or mean differences in SBP, DBP, BMI, total cholesterol, triglyceride, HDL-C, LDL-C and glucose.^12^ In the Japan Nurses’ Health Study, another cross-sectional study of 22 426 women, age at natural menopause (categorised as <45, 45 to 53 and >53) was not associated with risk of hypertension or diabetes, but early menopause was associated with risk of hypercholesterolaemia,^29^ which contrasts with our findings that showed no strong evidence of associations of age at period cessation with lipids in later life. Our findings showing that the type of period cessation (hysterectomy compared with natural menopause) was not associated with CVD risk factors are also comparable to findings from the SWAN^7^ and CARDIA^8^ studies, which were of similar design to ours and included repeated measures of risk factors across the menopausal transition. However, our study examines change across a wider age range and into the seventh decade of life, demonstrating that type and timing of period cessation are not associated with CVD risk factors once all women have passed through menopause and reach older ages where CVD is more common.

### Implications

If associations of age at period cessation and CVD events are causal and not the result of confounding in observational studies^1^ or shared genetic architecture of age at period cessation and CVD,^5^ our findings would suggest that changes in conventional CVD intermediates over the long term are an unlikely mediating pathway. The findings also have important implications for women and clinicians, as they suggest that any impact of age and type of period cessation on conventional CVD intermediates over the long term is likely to be small. However, studies with larger sample sizes in more heterogeneous populations are required to replicate our findings and provide more precise estimates of associations. Furthermore, some studies have shown that older age at period cessation may be associated with reduced risk of carotid atherosclerosis.^1^ Thus, there may still be associations with other CVD intermediates that have not been evaluated here, such as coronary artery calcification and vascular structure and function. 1 Further work examining age at period cessation and other CVD intermediates such as these may help to further elucidate the mechanisms underlying age at period cessation and CVD risk.

To aid interpretation, our findings should be considered in the context of the acute but transient changes in lipids observed close to the time of menopause in previous cohorts such as SWAN^6^ and the Women's Midlife Health Project.^30^ Although neither of these cohorts directly examined age at period cessation and change in CVD risk factors, it is possible that age at period cessation is associated with incident CVD risk not through a long-term effect on CVD risk factors but through acute effects on risk factors, which eventually attenuate over time. These acute effects close to the time of menopause may be clinically relevant for later CVD risk. Longitudinal cohorts examining age at period cessation and acute effects on intermediate risk factors close to the menopausal transition are required to provide a greater understanding of the aetiology of age at period cessation and CVD events, if reported associations in observational studies^1^ are causal.

### Strengths and limitations

There are several strengths to our study including the prospective, detailed and longitudinal collection of data on menopausal characteristics and CVD measures from 36 and 53 years. Our study is the first to our knowledge to examine the association of type and timing of period cessation with CVD risk factors into the seventh decade of life. We have included women who have undergone hysterectomy and who were taking HRT, which many previous analyses have excluded. We have captured the full range of ages at which menopause occurs, a strength over previous analyses which have often excluded women with very early or very late ages at period cessation. We have used multilevel models which take account of clustering of repeated measures within individuals and the correlation between measures over time, and performed several sensitivity analyses to examine the robustness of our findings to the effect of pharmacological treatment of risk factors. Limitations include combining non-fasting and fasting bloods for risk factors and the availability of measures from 36 years for only four out of the eight risk factors, as data for lipids and HbA1c were only available from the age of 53 years. Selection bias is also a potential limitation and individuals included in our analysis were more advantaged than those excluded due to missing exposure, outcome and confounder data, thus limiting the potential generalisability of our findings to the wider population.

## Conclusion

How and when women experience period cessation is unlikely to adversely affect conventional CVD intermediates from midlife. Women and clinicians concerned about the impact of type and timing of period cessation on conventional CVD intermediates from midlife should be reassured that the impact over the long term is likely to be small.

**Key messages**

**What is already known on this subject?**

- Age at period cessation is associated with cardiovascular disease (CVD). Whether age at period cessation adversely affects change in conventional CVD intermediates from mid to later life is not well understood.

**What might this study adds?**

- Using data from the 1946 British birth cohort study with prospective measurement of type and timing of period cessation and repeated measures of CVD risk factors from midlife to age 69 years, our findings suggest that the impact of type and timing of period cessation on conventional CVD intermediates from midlife is likely to be small over the long term.

**How might this impact on clinical practice?**

- Clinicians may be able to reassure women that the impact of type and timing of period cessation on conventional CVD risk factors such as blood pressure and lipids from midlife to age 69 years is likely to be small.
