# Supplementary material for: Age at period cessation and trajectories of cardiovascular risk factors across mid and later life
Source: Heart. 2020 Feb 25;106(7):499–505. doi: 10.1136/heartjnl-2019-315754 (PMC7079196; doi:10.1136/heartjnl-2019-315754)
Supplement: Supplementary data [file heartjnl-2019-315754supp001.pdf]

Online Supplemental Material

Age at period cessation and trajectories of cardiovascular risk factors across mid and later life

eAppendix 1 Details on confounders

We categorised participant's occupation as reported at age 53 into six classes (from professional to unskilled manual) according to the Registrar General social classification. Parity was self-reported by the participants at all data collections across adulthood and included as a continuous covariate. Women reported monthly histories of HRT use throughout follow-up and from this, HRT use (yes/no) as a time-varying covariate was included in our models. Age at menarche in years was obtained at medical examination by school doctors when participants were 14-15 years old, supplemented for those who had not reached menarche by 15 years (n=94) by retrospective reports of age at menarche by postal questionnaires when participants were aged 48 years [1]. Smoking, obtained from self-reported information at 36 years, was classified as former/current/never. Physical activity at 36 years, obtained from self-reports of frequency and duration of participation in leisure time activities was classified as inactive (reported no participation); moderately active (participated in relevant activities one to four times: in the previous month); or most active (participated in relevant activities five or more times: in the previous month [2].

eAppendix 2 Details of model selection

Models were derived by initially examining observed data for each risk factor and plotting mean values for each risk factor over time to examine the possible shape of the trajectory.

Linear spline multilevel models were used for SBP, DBP, WC and BMI. Based on the observed data for these, we compared observed and predicted measurements for a selection of suitable models for each risk factor. We examined rates of change between time periods in order to examine whether changes between periods were similar or different. In cases where rates of change between spline periods appeared identical, the fit of models with reduced splines was explored. Final models for SBP, DBP, BMI and WC had one knot placed at 53 years resulting in two periods of change; from 36-53 and from 53-69. This knot was selected based on examination of observed data over time and comparing model fit

statistics (Akaike's Information Criterion) for several models with different knot points (with knot points placed at whole years closest to mean age at clinics due to a greater density of measures). The selection of this knot point at age 53 years, also had the additional advantage of allowing comparability with the linear slopes modelled from 53 to 69 years for the blood-based biomarkers.

SBP and DBP

The models for SBP and DBP took the form of: SBP_{ij} /DBP_{ij} $= \beta_0 + u_{0j} + (\beta_1 + u_{1j})s_{ij1} + (\beta_2 + u_{2j})s_{ij2} + e_{ij}(age_{ij})$ where for person j at measurement occasion i; β_0 represents the fixed effect coefficient for the average intercept, β_1 to β_2 represent fixed effect coefficients for the average linear slopes of each linear spline, u_{0j} to u_{3j} indicate person-specific random effects for the intercept and slopes respectively, and e_{ij} represents the occasion-specific residuals or measurement error which was allowed to vary with age.

BMI, WC

BMI and WC were natural log transformed. The models for BMI and WC took the form of: log BMI_{ij} /log WC_{ij} $= \beta_0 + u_{0j} + (\beta_1 + u_{1j})s_{ij1} + (\beta_2 + u_{2j})s_{ij2} + e_{ij}(age_{ij})$ where for person j at measurement occasion i; β_0 represents the fixed effect coefficient for the average intercept, β_1 to β_3 represent fixed effect coefficients for the average linear slopes of each linear spline, u_{0j} to u_{2j} indicate person-specific random effects for the intercept and slopes respectively, and e_{ij} represents the occasion-specific residuals or measurement error which was allowed to vary with age.

Lipids and HBA1c

Triglyceride and glycated haemoglobin were natural log transformed. Lipids and HBA1c were modelled using a linear age term.The models for lipids and HBA1C took the form of: log triglycerides $_{ij}$ / $LDL\text{-}C_{ij}$ /$HDL\text{-}C_{ij}$ / log HBA1c $_{ij} = \beta_0 + u_{0j} + (\beta_1 + u_{1j})\, age_{ij1} + e_{ij}(age_{ij})$ where for person j at measurement occasion i; β_0 represents the fixed effect coefficient for the average intercept, β_1 represents fixed effect coefficients

for the average linear slope, u_{0j} and u_{1j} indicate person-specific random effects for the intercept and slopes respectively, and e_{ij} represents the occasion-specific residuals or measurement error which was allowed to vary with age.

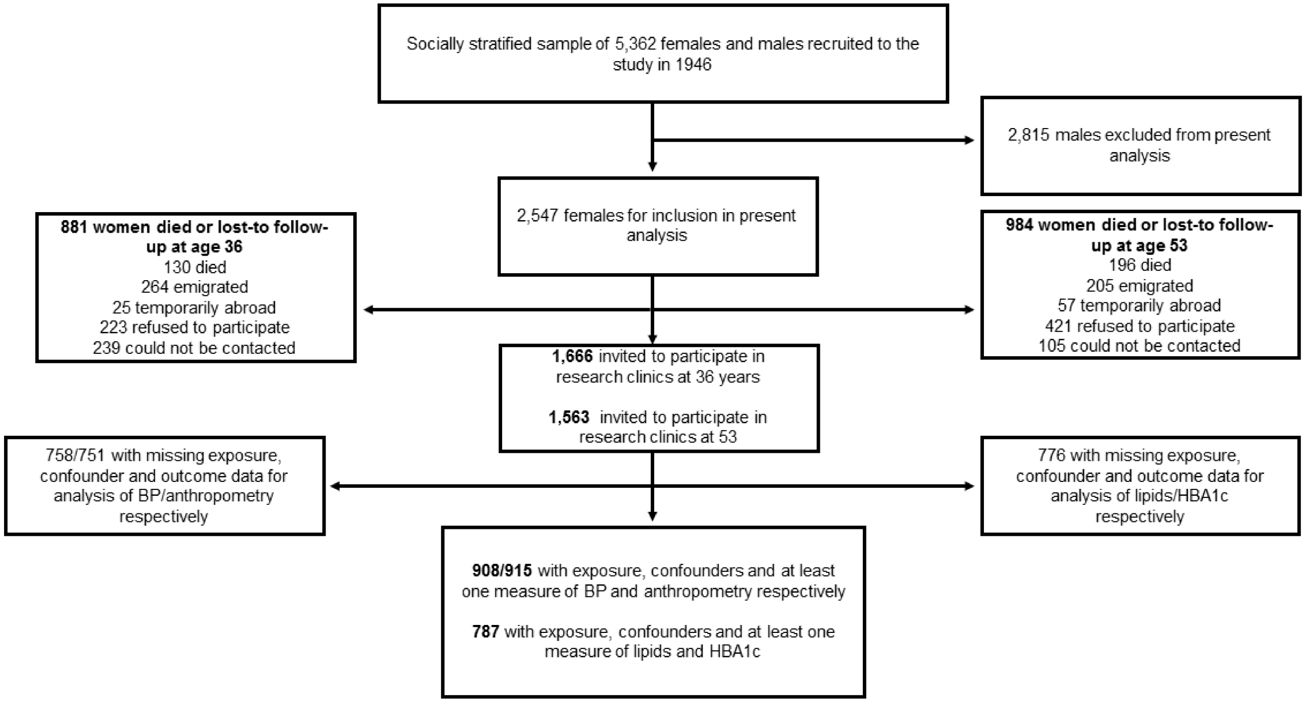

eFigure 1 Flow diagram of participants included in study

BP, blood pressure; HBA1c, glycated haemoglobin.

eTable 1 Number of participants with cardiometabolic measures at each time point

	Age 36	Age 43	Age 53	Age 60-64	Age 69	Total measures	Total participants	Median measures (IQR)
**Lipids/HBA1c†**			575	555	520	1650	787	3 (2-3)
**BMI/WC**	887	858	692	677	637	3751	915	5 (4-5)
**SBP/DBP**	887	851	686	671	633	3728	908	5 (4-5)

DBP, diastolic blood pressure; HBA1C; glycated haemoglobin; HDL-C, high density lipoprotein cholesterol; IQR, interquartile range; LDL-C, low density lipoprotein cholesterol; SBP, systolic blood pressure.

† Triglyceride, HDL-C and LDL-C.

eTable 2 Model details for lipid and log HBA1c trajectories

	No of contributing individuals		Assessment of model fit			
	Total number of observations	Number of individuals with 1 measure	Mean observed, (SD)*	Mean predicted, (SD)*	Mean difference (observed – predicted) *	95% level of agreement between observed and predicted*
**Log triglyceride**						
**Overall**	1650	787				
53 years	575	575	0.39 (0.47)	0.32 (0.29)	0.0624	-0.49,0.62
53 -64 years	963	684	0.26 (0.50)	0.30 (0.28)	-0.0344	-0.64,0.57
64-69 years	686	576	0.29 (0.46)	0.24 (0.24)	0.0474	-0.58,0.67
**HDL-C**						
**Overall**	1650	787				
53 years	575	575	1.86 (0.48)	1.84 (0.35)	0.0159	-0.35,0.38
53 -64 years	963	684	1.81 (0.46)	1.82 (0.35)	-0.0062	-0.36,0.35
64-69 years	686	576	1.77 (0.46)	1.76 (0.37)	0.0087	-0.31,0.33
**LDL-C**						
**Overall**	1650	787				
53 years	575	575	3.56 (1.01)	3.66 (0.47)	-0.1000	-1.46,1.26
53 -64 years	963	684	3.63 (1.03)	3.58 (0.50)	0.0582	-1.32,1.44
64-69 years	686	576	3.19 (1.02)	3.28 (0.51)	-0.0820	-1.36,1.19
**Log HBA1c**						
**Overall**	1650	787				
53 years	575	575	3.62 (0.14)	3.63 (0.10)	-0.0054	-0.12,0.11
53 -64 years	963	684	3.64 (0.13)	3.64 (0.10)	0.0032	-0.10,0.11
64-69 years	686	576	3.67 (0.12)	3.67 (0.10)	-0.0045	-0.10,0.09

HBA1c, glycated haemoglobin; HDL-C, high density lipoprotein cholesterol; LDL-C, low density lipoprotein cholesterol.

* Triglyceride and HBA1c are natural log transformed. All values are in log form.

eTable 3 Model details for blood pressure and anthropometry trajectories

	No of contributing individuals		Assessment of model fit			
	Total number of observations	Number of individuals with 1 measure	Mean observed, (SD)*	Mean predicted, (SD)*	Mean difference (observed – predicted) *	95% level of agreement between observed and predicted*
**SBP**						
**Overall**	3728	908				
36 years	887	887	116.90 (13.61)	115.97 (5.32)	0.6125	-19.19,20.41
36 -53 years	1739	905	119.05 (14.95)	119.29 (7.06)	-0.4514	-22.10,21.20
53-69 years	1989	837	132.64 (17.85)	132.37 (9.88)	0.2714	-22.44,22.98
**DBP**						
**Overall**	3728	908				
36 years	887	887	74.77 (11.53)	74.43 (3.98)	0.2263	-17.80,18.25
36 -53 years	1739	905	75.81 (11.81)	75.90 (4.37)	-0.1485	-18.57,18.27
53-69 years	1989	837	77.19 (10.92)	77.08 (5.53)	0.0956	-15.68,15.87
**Log BMI**						
**Overall**	3751	915				
36 years	887	887	3.15 (0.15)	3.14 (0.14)	0.0019	-0.06,0.07
36 -53 years	1746	912	3.18 (0.16)	3.18 (0.15)	-0.0009	-0.08,0.08
53-69 years	2005	844	3.32 (0.19)	3.32 (0.17)	0.0004	-0.07,0.07
**Log WC**						
**Overall**	3751	915				
36 years	887	887	4.33 (0.14)	4.31 (0.09)	0.0131	-0.11,0.14
36 -53 years	1746	912	4.34 (0.14)	4.34 (0.10)	-0.0060	-0.14,0.12
53-69 years	2003	844	4.49 (0.15)	4.49 (0.13)	0.0043	-0.10,0.11

DBP, diastolic blood pressure; SBP, systolic blood pressure; WC, waist circumference.

*BMI and WC are natural log transformed. All values are in log form.

eAppendix 3 Additional and sensitivity analyses

We examined the characteristics of participants included in analyses of anthropometry compared with those excluded due to missing exposure, outcome or confounder data or loss-to follow-up to better understand the role of selection bias. We examined whether pharmacologic treatment of blood pressure, lipids and HBA1c could have influenced our findings by adding a range of constant values to the risk factor measurements of any individual reporting being on treatment at each time point. We performed sensitivity analyses adding 20%, 40% and 60% to the triglyceride values of individuals reporting treatment with lipid lowering medication at each time point, 10%, 20% and 30% to LDL-C values of participants reporting treatment with lipid lowering medication at each time point, and subtracting 5%, 10% and 15% from the HDL-C values of individuals under treatment at each time point. For HBA1c, we performed separate analyses adding 1%, 2% and 3% to the values of individuals reporting treatment with diabetes medications at each time point. Similarly, we examined the potential effect of treatment with antihypertensive medications on our findings, adding 10%, 20% and 30% to the recorded SBP of participants under treatment at each time point, and 5%, 10% and 15% to the recorded DBP of participants under treatment.

In order to examine whether findings differed by type of hysterectomy, we tested whether the associations of age at period cessation with cardiovascular risk factors differed between women who had hysterectomy with bilateral oophorectomy compared with hysterectomy with conservation of at least one ovary. Trajectories of risk factors were examined in women who could have been pre- or post-menopausal at age 53. Therefore, we performed a sensitivity analysis excluding women who were still pre-menopausal at 53 years, to understand if our findings differed when the analyses were restricted to post-menopausal measures of cardiovascular risk factors.

eTable 4 Characteristics of women included in primary analyses of anthropometry compared to women excluded from analyses due to missing data

	Included in analysis (N=915)	Excluded from analysis (N=448 – 1141)
	**n (%)**	**n (%)**
**Household social class**		
Skilled (non-manual)	332 (36.3)	435 (38.1)
Professional/intermediate	321 (35.1)	353 (30.9)
Skilled manual and partly skilled	198 (22.1)	302 (26.5)
Unskilled	60 (6.6)	51 (4.5)
**Parity**		
0	115 (12.6)	72 (11.9)
1 or 2	509 (55.6)	347 (57.5)
3 or more	291 (31.8)	185 (30.6)
**Current smoking at age 36**	276 (30.1)	282 (37.6)
**Physical activity at age 36**		
Inactive	385 (42.1)	320 (42.8)
Less active	220 (24.0)	182 (24.3)
Most active	310 (33.9)	246 (32.9)
**HRT use**		
Age 36	9 (1.0)	7 (0.9)
Age 43	32 (3.7)	40 (5.2)
Age 53	193 (27.9)	239 (46.0)
Age 60-64	32 (4.7)	48 (10.4)
Age 69	16 (2.5)	22 (4.5)
**Type of menopause**		
Natural	675 (73.7)	577 (80.8)
Hysterectomy	240 (26.2)	137 (19.2)
	**Mean (SD)**	**Mean (SD)**
**Mean age at menarche (SD)**	13.0 (1.2)	13.1 (1.1)
**Mean age at period cessation (SD)**	49.6 (5.6)	49.5 (6.1)
**Mean BMI at age 36 (SD)**	23.6 (3.9)	23.5 (4.2)
**Mean SBP at age 36 (SD)**	116.9 (13.6)	17.3 (14.6)

BMI, body mass index; SBP, systolic blood pressure; SD, standard deviation. Denominator for participants excluded may vary due to missing data on the characteristics included in the table.

eTable 5 Unadjusted association of age at period cessation (per year increase) with anthropometry and blood pressure from 36 to 69 years

	Mean trajectory for NM	Mean difference in trajectory per year increase in age at NM	Mean trajectory for HY	Mean difference in trajectory per year increase in HY	P value for interaction of type and timing of menopause*
**SBP**					
Age 36	116.0 (114.8,117.2)	0.02(-0.26,0.30)	115.1 (112.8,117.5)	-0.06(-0.36,0.24)	0.91
Δ 36- 53	0.90(0.79,1.01)	0.01(-0.01,0.04)	1.00(0.78,1.22)	0.01(-0.01,0.04)	0.45
Δ 53 -69	0.18(0.06,0.30)	-0.03(-0.05,-0.003)	0.09(-0.14,0.31)	-0.01(-0.04,0.02)	0.08
Age 69	134.1 (132.5,135.8)	-0.22(-0.59,0.14)	133.5 (130.4,136.6)	-0.02(-0.39,0.35)	0.30
**DBP**					
Age 36	74.3(73.4,75.2)	0.001(-0.20,0.20)	74.9(73.2,76.6)	0.06(-0.14,0.27)	0.84
Δ 36- 53	0.41(0.33,0.48)	0.01(-0.01,0.02)	0.40(0.25,0.55)	0.0002(-0.02,0.02)	0.83
Δ 53 -69	-0.46(-0.54,-0.38)	-0.01(-0.03,0.01)	-0.46(-0.61,-0.30)	-0.01(-0.03,0.01)	0.37
Age 69	73.9(72.9,74.8)	-0.08(-0.30,0.14)	74.4(72.5,76.2)	-0.03(-0.26,0.20)	0.59
**Log BMI**					
Age 36	3.14(3.13,3.15)	0.002(-0.001,0.005)	3.15(3.12,3.17)	-0.003(-0.01,0.00001)	0.13
Δ 36- 53	0.01(0.01,0.01)	0.0001(-0.0001,0.0002)	0.01(0.01,0.01)	-0.00001(-0.0002,0.0002)	0.54
Δ 53 -69	0.002(0.002,0.003)	-0.00004(-0.0002,0.0001)	0.002(0.001,0.004)	-0.0001(-0.0003,0.0001)	0.39
Age 69	3.33(3.31,3.34)	0.002(-0.002,0.007)	3.35(3.32,3.39)	-0.005(-0.01,-0.0009)	0.04
**Log WC**					
Age 36	4.31(4.30,4.32)	0.002(-0.0003,0.005)	4.32(4.30,4.34)	-0.001(-0.003,0.002)	0.20
Δ 36- 53	0.007(0.007,0.008)	-0.0001(-0.0002,0.0001)	0.007(0.006,0.009)	-0.00007(-0.0002,0.0001)	0.43
Δ 53 -69	0.006(0.005,0.007)	0.0001(-0.0001,0.0003)	0.006(0.004,0.007)	-0.0001(-0.0002,0.0001)	0.43
Age 69	4.53(4.52,4.55)	0.002(-0.0010,0.01)	4.53(4.51,4.56)	-0.003(-0.006,0.0002)	0.05

Legend: DBP, diastolic blood pressure; log WC, natural log waist circumference; HY, hysterectomy; NM, natural menopause; SBP, systolic blood pressure. Note that BMI and WC are natural log transformed and all values presented are in log form. Δ = change per year in risk factor.
* P value for the interaction of age and type at period cessation with trajectories.

eTable 6 Association of age at period cessation (per year increase) with anthropometry and blood pressure from 36 to 69 years, adjusted for co-variates

	Mean trajectory for NM (reference for period cessation at age 50)	Mean difference in trajectory per year increase in age at NM	Mean trajectory for HY (reference for period cessation at age 50)	Mean difference in trajectory per year increase in HY	P value for interaction of type and timing of menopause*
**SBP**					
Age 36	119.0(115.5,122.4)	0.08(-0.20,0.36)	118.2(113.9,122.5)	-0.07(-0.38,0.24)	0.80
Δ 36- 53	0.53(0.21,0.85)	0.01(-0.02,0.03)	0.56(0.16,0.96)	0.01(-0.02,0.04)	0.63
Δ 53 -69	0.44(0.10,0.77)	-0.02(-0.05,0.003)	0.37(-0.05,0.79)	-0.01(-0.04,0.02)	0.18
Age 69	135.0(130.2,139.7)	-0.16(-0.52,0.20)	133.7(127.9,139.4)	-0.04(-0.40,0.33)	0.48
**DBP**					
Age 36	75.7(73.2,78.2)	0.03(-0.17,0.23)	76.2(73.1,79.3)	0.09(-0.12,0.30)	0.69
Δ 36- 53	0.3(0.08,0.52)	0.003(-0.01,0.02)	0.30(0.02,0.57)	0.002(-0.02,0.02)	0.93
Δ 53 -69	-0.40(-0.64,-0.17)	-0.01(-0.03,0.01)	-0.41(-0.70,-0.12)	-0.01(-0.03,0.01)	0.42
Age 69	74.3(71.4,77.2)	-0.07(-0.28,0.15)	74.7(71.2,78.2)	-0.08(-0.30,0.15)	0.68
**Log BMI**					
Age 36	3.16(3.12,3.19)	0.002(-0.001,0.005)	3.16(3.11,3.20)	-0.003(-0.005,0.0006)	0.09
Δ 36- 53	0.008(0.006,0.01)	0.0001(-0.0001,0.0002)	0.009 (0.006,0.01)	0.00001(-0.0001,0.0002)	0.64
Δ 53 -69	0.004(0.002,0.006)	-0.00003(-0.0002,0.0001)	0.003(0.001,0.006)	-0.0001(-0.0003,0.0001)	0.44
Age 69	3.35(3.30,3.40)	0.003(-0.001,0.007)	3.36(3.30,3.42)	-0.004(-0.008,-0.0003)	0.02
**Log WC**					
Age 36	4.32(4.29,4.35)	0.003(0.0004,0.005)	4.33(4.29,4.36)	-0.001(-0.003,0.002)	0.07
Δ 36- 53	0.007(0.005,0.009)	-0.0001(-0.0003,0.0001)	0.007(0.005,0.010)	-0.00004(-0.0002,0.0001)	0.46
Δ 53 -69	0.008(0.006,0.010)	0.0001(-0.0001,0.0003)	0.007(0.004,0.010)	-0.0001(-0.0002,0.0001)	0.34
Age 69	4.57(4.53,4.61)	0.003(-0.0002,0.01)	4.56(4.52,4.61)	-0.002(-0.005,0.001)	0.02

Legend: DBP, diastolic blood pressure; log WC, natural log waist circumference; HY, hysterectomy; NM, natural menopause; SBP, systolic blood pressure. Note that BMI and WC are natural log transformed and all values presented are in log form. Δ = change per year in risk factor.
* P value for the interaction of age and type at period cessation with trajectories.
Trajectories adjusted for socioeconomic position, parity, time-varying hormone replacement therapy use, age at menarche, BMI at age 36 (SBP and DBP only), smoking at age 36, physical activity at age 36.

eTable 7 Unadjusted association of age at period cessation (per year increase) with blood markers from 53 to 69 years

	Mean trajectory for NM	Mean difference in trajectory per year increase in age at NM	Mean trajectory for HY	Mean difference in trajectory per year increase in HY	P value for interaction of type and timing of menopause*
**Log trig**					
Age 53	0.31(0.26,0.36)	-0.004(-0.02,0.01)	0.36(0.26,0.46)	-0.01(-0.03,0.001)	0.15
Δ53-69	-0.005(-0.01,-0.001)	0.0003(-0.0005,0.001)	-0.01(-0.01,-0.0003)	0.0002(-0.001,0.001)	0.63
Age 69	0.24(0.19,0.28)	0.001(-0.01,0.01)	0.20(0.09,0.32)	-0.01(-0.02,0.002)	0.32
**LDL-C**					
Age 53	3.71(3.60,3.81)	-0.02(-0.04,0.01)	3.66(3.45,3.87)	0.01(-0.02,0.04)	0.34
Δ53-69	-0.02(-0.03,-0.01)	0.0003(-0.002,0.002)	-0.03(-0.04,-0.01)	0.0001(-0.002,0.002)	0.95
Age 69	3.34(3.23,3.44)	-0.01(-0.03,0.01)	3.30(3.02,3.57)	0.01(-0.01,0.03)	0.24
**HDL-C**					
Age 53	1.82(1.78,1.87)	-0.002(-0.01,0.01)	1.86(1.78,1.95)	-0.001(-0.01,0.01)	0.93
Δ53-69	-0.003(-0.01,-0.001)	-0.0003(-0.001,0.0003)	-0.01(-0.01,-0.004)	0.001(-0.0001,0.001)	0.18
Age 69	1.77(1.72,1.81)	-0.007(-0.02,0.003)	1.67(1.58,1.77)	0.01(-0.002,0.02)	0.07
**Log HBA1c**					
Age 53	3.64(3.63,3.66)	-0.003(-0.006,0.00004)	3.62(3.59,3.64)	-0.002(-0.005,0.001)	0.08
Δ53-69	0.002(0.001,0.003)	0.0003(0.0002,0.0005)	0.004(0.003,0.006)	-0.00001(-0.0002,0.0002)	0.001
Age 69	3.67(3.66,3.69)	0.002(-0.0005,0.005)	3.71(3.68,3.74)	-0.002(-0.005,0.0011)	0.042

Legend: HBA1c, glycated haemoglobin; HDL-C, high density lipoprotein cholesterol; HY, hysterectomy; LDL-C, low density lipoprotein cholesterol; NM, natural menopause Note that triglyceride and HBA1c are natural log transformed. Δ = change per year in risk factor.
* P value for the interaction of age and type at period cessation with trajectories.

eTable 8 Association of age at period cessation (per year increase) with blood markers from 53 to 69 years, adjusted for co-variates

	Mean trajectory for NM (reference for period cessation at age 50)	Mean difference in trajectory per year increase in age at NM	Mean trajectory for HY (reference for period cessation at age 50)	Mean difference in trajectory per year increase in HY	P value for interaction of type and timing of menopause *
**Log trig**					
Age 53	0.46(0.31,0.60)	-0.005(-0.02,0.01)	0.44(0.26,0.62)	-0.01(-0.02,0.005)	0.23
Δ53-69	-0.01(-0.02,0.005)	0.0004(-0.0004,0.001)	-0.003(-0.02,0.01)	0.0001(-0.001,0.001)	0.52
Age 69	0.37(0.24,0.50)	0.002(-0.01,0.01)	0.41(0.23,0.59)	-0.006(-0.02,0.004)	0.34
**LDL-C**					
Age 53	3.89(3.59,4.20)	-0.01(-0.04,0.01)	4.03(3.65,4.41)	0.01(-0.02,0.04)	0.38
Δ53-69	-0.03(-0.06,-0.01)	0.0002(-0.002,0.002)	-0.04(-0.07,-0.01)	-0.001(-0.003,0.001)	0.85
Age 69	3.40(3.10,3.70)	-0.01(-0.03,0.01)	3.21(2.80,3.62)	-0.002(-0.02,0.02)	0.36
**HDL-C**					
Age 53	1.67(1.55,1.79)	-0.002(-0.012,0.008)	1.70(1.54,1.85)	-0.005(-0.015,0.005)	0.75
Δ53-69	0.006(-0.003,0.014)	-0.0003(-0.0009,0.0004)	0.003(-0.007,0.013)	0.0007(0.0001,0.0013)	0.14
Age 69	1.76(1.63,1.90)	-0.007(-0.017,0.004)	1.72(1.55,1.88)	0.007(-0.003,0.016)	0.09
**Log HBA1c**					
Age 53	3.68(3.65,3.72)	-0.003(-0.005,0.0004)	3.67(3.63,3.72)	-0.0008(-0.004,0.002)	0.17
Δ53-69	-0.0001(-0.002,0.002)	0.0003(0.0001,0.0005)	0.001(-0.002,0.004)	-0.00002(-0.0002,0.0002)	0.003
Age 69	3.68(3.64,3.72)	0.0024(-0.0005,0.005)	3.69(3.65,3.74)	-0.001(-0.004,0.002)	0.052

Legend: HBA1c, glycated haemoglobin; HDL-C, high density lipoprotein cholesterol; HY, hysterectomy; LDL-C, low density lipoprotein cholesterol; NM, natural menopause Note that triglyceride and HBA1c are natural log transformed and all values are in log form. Δ = change per year in risk factor.

* P value for the interaction of age and type at period cessation with trajectories.

Adjusted for socioeconomic position, parity, time-varying hormone replacement therapy use, age at menarche, BMI at age 36, smoking at age 36, physical activity at age 36.

eTable 9 Association of type of menopause with blood pressure and anthropometry from 36 to 69 years

	Unadjusted			Adjusted		
	**Mean NM (reference)**	**Association of HY**	**P***	**Mean NM (reference)**	**Association of HY**	**P***
**SBP**						
Age 36	116.0(114.8,117.2)	-0.85(-3.51,1.81)	0.53	119.0(115.5,122.4)	-0.77(-3.45, 1.90)	0.57
Δ 36- 53	0.90(0.79,1.01)	0.10(-0.14,0.35)	0.42	0.53(0.21,0.85)	0.03(-0.22,0.28)	0.80
Δ 53 -69	0.18(0.06,0.30)	-0.09(-0.35,0.16)	0.47	0.44(0.10,0.77)	-0.07(-0.33,0.19)	0.62
Age 69	134.1(132.5,135.8)	-0.62(-4.13,2.89)	0.73	135.0(130.2,139.7)	-1.28(-4.81,2.25)	0.48
**DBP**						
Age 36	74.3(73.4,75.2)	0.61(-1.33,2.54)	0.54	75.7(73.2,78.2)	0.55(-1.39,2.50)	0.58
Δ 36- 53	0.41(0.33,0.48)	-0.01(-0.18,0.16)	0.92	0.3(0.08,0.52)	-0.003(-0.18,0.17)	0.97
Δ 53 -69	-0.46(-0.54,-0.38)	0.003 (-0.17,0.18)	0.97	-0.40(-0.64,-0.17)	-0.002(-0.18,0.18)	0.98
Age 69	73.9(72.9,74.8)	0.51(-1.60,2.63)	0.63	74.3(71.4,77.2)	0.44(-1.69,2.57)	0.68
**Log BMI**						
Age 36	3.14(3.13,3.15)	0.01(-0.02,0.04)	0.54	3.16(3.12,3.19)	0.003(-0.025,0.031)	0.84
Δ 36- 53	0.009(0.008,0.010)	0.001(-0.0005,0.002)	0.18	0.008(0.006,0.010)	0.001(-0.0004,0.003)	0.16
Δ 53 -69	0.002(0.002,0.003)	-0.00003(-0.0016,0.0015)	0.97	0.004(0.002,0.006)	-0.0003(-0.002,0.001)	0.73
Age 69	3.33(3.31,3.34)	0.03(-0.01,0.06)	0.19	3.35(3.30,3.40)	0.02(-0.02,0.05)	0.38
**Log WC**						
Age 36	4.31(4.30,4.32)	0.01(-0.01,0.03)	0.41	4.32(4.29,4.35)	0.002(-0.021,0.026)	0.84
Δ 36- 53	0.007(0.007,0.008)	-0.0004(-0.0019,0.0012)	0.67	0.007(0.005,0.009)	-0.00005 (-0.0016,0.0017)	0.99
Δ 53 -69	0.006(0.005,0.007)	-0.0003(-0.0019,0.0013)	0.69	0.008(0.006,0.010)	-0.0005(-0.0022,0.0011)	0.52
Age 69	4.53(4.52,4.55)	0.00(-0.03,0.03)	0.95	4.57(4.53,4.61)	-0.01(-0.04,0.02)	0.70

Legend: DBP, diastolic blood pressure; log WC, log waist circumference; HY, hysterectomy; NM, natural menopause; SBP, systolic blood pressure. Note that BMI and WC are natural log transformed and all values presented are in log form. Δ = change per year in risk factor.

*P value for association of type of menopause (HY compared with NM) with trajectory.

Adjusted for socioeconomic position, parity, age at period cessation, time-varying hormone replacement therapy use, age at menarche, BMI at age 36 (SBP and DBP only), smoking and physical activity at age 36.

eTable 10 Association of type of menopause with blood markers from 53 to 69 years

	Unadjusted			Adjusted		
	**Mean NM (reference)**	**Association of HY**	**P***	**Mean NM (reference)**	**Association of HY**	**P***
**Log triglyceride**						
Age 53	0.31(0.26,0.36)	0.05(-0.07,0.16)	0.41	0.46(0.31,0.60)	-0.02(-0.13,0.10)	0.77
Δ 53-69	-0.005(-0.008,-0.001)	-0.002(-0.009,0.005)	0.58	-0.005(-0.016,0.005)	0.003(-0.005,0.010)	0.52
Age 69	0.24(0.19,0.28)	0.01(-0.08,0.11)	0.77	0.37(0.24,0.50)	0.02(-0.07,0.12)	0.63
**LDL-C**						
Age 53	3.71(3.60,3.81)	-0.05(-0.28,0.19)	0.69	3.89(3.59,4.20)	0.13(-0.11,0.38)	0.28
Δ 53-69	-0.02(-0.03,-0.01)	-0.002(-0.020,0.015)	0.79	-0.03(-0.06,-0.01)	-0.012(-0.030,0.007)	0.21
Age 69	3.34(3.23,3.44)	-0.09(-0.30,0.13)	0.43	3.40(3.10,3.70)	-0.05(-0.26,0.16)	0.64
**HDL-C**						
Age 53	1.82(1.78,1.87)	0.04(-0.06,0.14)	0.40	1.70 (1.55,1.85)	0.03(-0.07,0.12)	0.59
Δ 53-69	-0.003(-0.006,-0.001)	-0.0060(-0.0120,-0.0001)	0.05	0.003(-0.003,0.014)	-0.003(-0.009,0.003)	0.33
Age 69	1.77(1.72,1.81)	-0.05(-0.15,0.04)	0.28	1.72(1.65,1.88)	-0.02(-0.12,0.07)	0.65
**Log HBA1c**						
Age 53	3.64(3.63,3.66)	-0.03(-0.06,0.001)	0.06	3.68(3.65,3.72)	-0.01(-0.04,0.02)	0.55
Δ 53-69	0.002(0.001,0.003)	0.002(0.001,0.004)	0.01	0.000(-0.002,0.002)	0.001(-0.001,0.002)	0.37
Age 69	3.67(3.66,3.69)	0.01(-0.02,0.04)	0.58	3.68(3.64,3.72)	0.004 (-0.02,0.03)	0.78

Legend: HBA1c, glycated haemoglobin; HDL-C, high density lipoprotein cholesterol; HY, hysterectomy; LDL-C, low density lipoprotein cholesterol; NM, natural menopause Note that triglyceride and HBA1c are natural log transformed and all values are in log form. Δ = change per year in risk factor.
*P value for association of type of menopause (HY compared with NM) with trajectory.
Adjusted for socioeconomic position, parity, age at period cessation, time-varying hormone replacement therapy use, age at menarche, BMI at age 36, smoking and physical activity at age 36.

1. Cooper R, Blell M, Hardy R, et al. Validity of age at menarche self-reported in adulthood. *Journal of epidemiology and community health.* 2006;60(11):993-997.
2. Cooper R, Mishra GD, Kuh D. Physical activity across adulthood and physical performance in midlife: findings from a British birth cohort. *American journal of preventive medicine.* 2011;41(4):376-384.
